# The negative feedback loop of NF-κB/miR-376b/NFKBIZ in septic acute kidney injury

**DOI:** 10.1172/jci.insight.142272

**Published:** 2020-12-17

**Authors:** Zhiwen Liu, Chengyuan Tang, Liyu He, Danyi Yang, Juan Cai, Jiefu Zhu, Shaoqun Shu, Yuxue Liu, Lijun Yin, Guochun Chen, Yu Liu, Dongshan Zhang, Zheng Dong

**Affiliations:** 1 Department of Nephrology and; 2 Department of Emergency Medicine, The Second Xiangya Hospital at Central South University, Changsha, Hunan, China.; 3 Department of Cellular Biology and Anatomy, Medical College of Georgia at Augusta University and Charlie Norwood VA Medical Center, Augusta, Georgia, USA.

**Keywords:** Nephrology, Diagnostics, Epigenetics, NF-kappaB

## Abstract

Sepsis is the leading cause of acute kidney injury (AKI). However, the pathogenesis of septic AKI remains largely unclear. Here, we demonstrate a significant decrease of microRNA-376b (miR-376b) in renal tubular cells in mice with septic AKI. Urinary miR-376b in these mice was also dramatically decreased. Patients with sepsis with AKI also had significantly lower urinary miR-376b than patients with sepsis without AKI, supporting its diagnostic value for septic AKI. LPS treatment of renal tubular cells led to the activation of NF-κB, and inhibition of NF-κB prevented a decrease of miR-376b. ChIP assay further verified NF-κB binding to the miR-376b gene promoter upon LPS treatment. Functionally, miR-376b mimics exaggerated tubular cell death, kidney injury, and intrarenal production of inflammatory cytokines, while inhibiting miR-376b afforded protective effects in septic mice. Interestingly, miR-376b suppressed the expression of NF-κB inhibitor ζ (NFKBIZ) in both in vitro and in vivo models of septic AKI. Luciferase microRNA target reporter assay further verified NFKBIZ as a direct target of miR-376b. Collectively, these results illustrate the NF-κB/miR-376b/NFKBIZ negative feedback loop that regulates intrarenal inflammation and tubular damage in septic AKI. Moreover, urinary miR-376b is a potential biomarker for the diagnosis of AKI in patients with sepsis.

## Graphical abstract

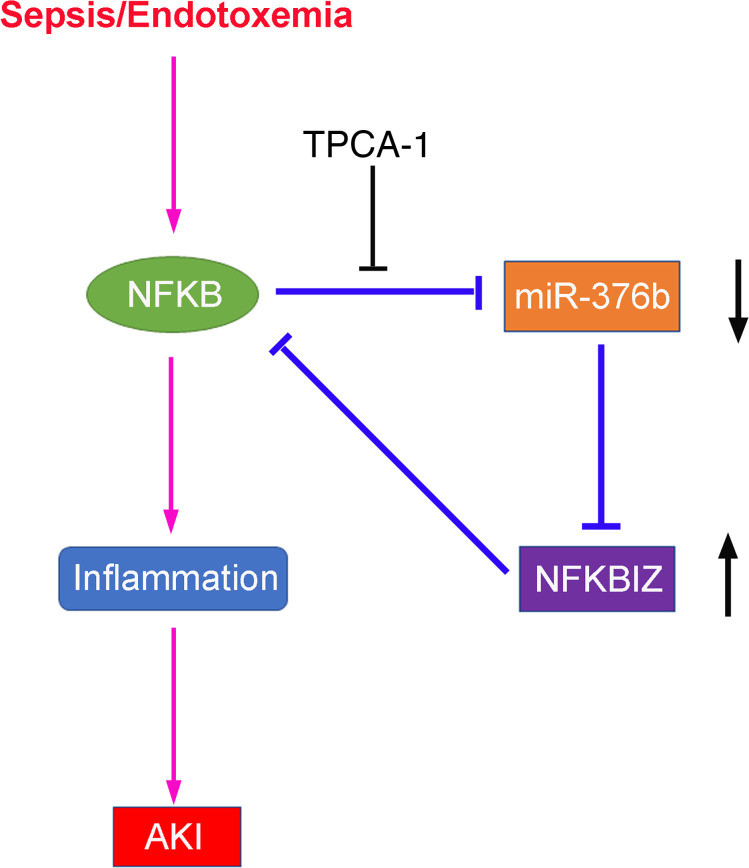

## Introduction

Sepsis is a serious disease caused by a dysregulated host response to infection accompanied by acute organ failure. The kidneys are often among the first organs to be affected by sepsis, resulting in nearly 50% of cases of acute kidney injury (AKI) in intensive care units. Unfortunately, septic AKI is associated with a high mortality rate (1, 2). The pathogenesis of septic AKI is complex and apparently multifactorial, involving renal tubular, microvascular, and inflammatory mechanisms that may interplay with and amplify each other (3–10). However, these mechanisms remain largely unclear, and effective treatment and specific diagnostic biomarkers for septic AKI are not available.

Inflammation plays a critical role in septic AKI (1, 11–14). Upon sepsis, circulating inflammatory cytokines can directly cause kidney tissue damage. Meanwhile, rather than a mere victim of inflammation, renal tubular cells become the propagator of intrarenal inflammation in septic AKI (1, 12). In this regard, LPSs and other pathogen-associated molecular patterns (PAMPs) can directly interact with TLR2 and TLR4 on renal tubule cells to activate intracellular inflammation pathways for cytokine production. These cytokines further induce leukocyte extravasation and infiltration into the kidneys, resulting in a vicious cycle of inflammation and tissue damage (2, 12).

NF-κB is a key to the proinflammatory response in a variety of cell types, including renal resident cells (15–17). The NF-κB family is composed of 5 related protein members, p50, p52, RelA (p65), RelB, and c-Rel, which are mainly regulated by IκBs and IκB kinase (IKK). Normally, IκBs bind with NF-κB to mask their nuclear localization signals and keep them sequestered in the cytoplasm. In response to various cytokines, inflammatory molecules, and stress signals, IKKs are activated by phosphorylate IκB, resulting in the release and translocation of NF-κB into the nucleus for the transcription of various downstream genes. Interestingly, the downstream genes include IκB, i.e., the inhibitor of NF-κB, forming a feedback loop for autoregulation of NF-κB and associated inflammation (18). NF-κB has been implicated in the pathogenesis of AKI (15, 16), but its regulation and function in septic AKI remains elusive.

miRNAs are small noncoding RNA molecules composed of approximately 22 nucleotides. In mammalian cells, miRNAs repress gene expression mainly by binding to the 3′-UTRs of their target gene mRNA, thereby blocking their translation. mRNAs are known to regulate animal development, physiology, and the pathogenesis of various diseases (19). In kidneys, microRNAs have been implicated in renal development, physiology, and disease pathogenesis (20–26). In AKI, ablation of Dicer (a key gene for microRNA biogenesis) in kidney proximal tubules led to resistance of mice to renal ischemia/reperfusion injury, suggesting a critical role of miRNAs in ischemic AKI (27). Follow-up studies identified specific miRNAs that play crucial roles in ischemic as well as nephrotoxic AKI (23, 25). Although specific miRNAs may contribute to septic AKI, the role of miRNAs in septic AKI is less clear. For example, compared with WT mice, microRNA-223–knockout (miR-223–knockout) mice were more resistant to septic AKI induced by cecal ligation and puncture (CLP) but they were more sensitive to LPS-induced endotoxic AKI, suggesting a complex role of this miRNA in septic AKI (28).

In this study, we have demonstrated that in septic AKI NF-κB is activated to suppress miR-376b in renal tubular cells. Interestingly, we have further identified NF-κB inhibitor ζ (NFKBIZ) as a direct target of miR-376b. Thus, suppression of miR-376b by NF-κB in septic AKI results in the upregulation of NFKBIZ, providing a negative feedback mechanism to contain NF-κB activation and associated inflammation. Furthermore, we have demonstrated a significant decrease of urinary miR-376b in both experimental models of septic AKI and in patients with sepsis and AKI, suggesting the potential of urinary miR-376b as a biomarker for septic AKI.

## Results

### miR-376b is downregulated in renal tubular cells during LPS-induced AKI.

**Figure 1:**
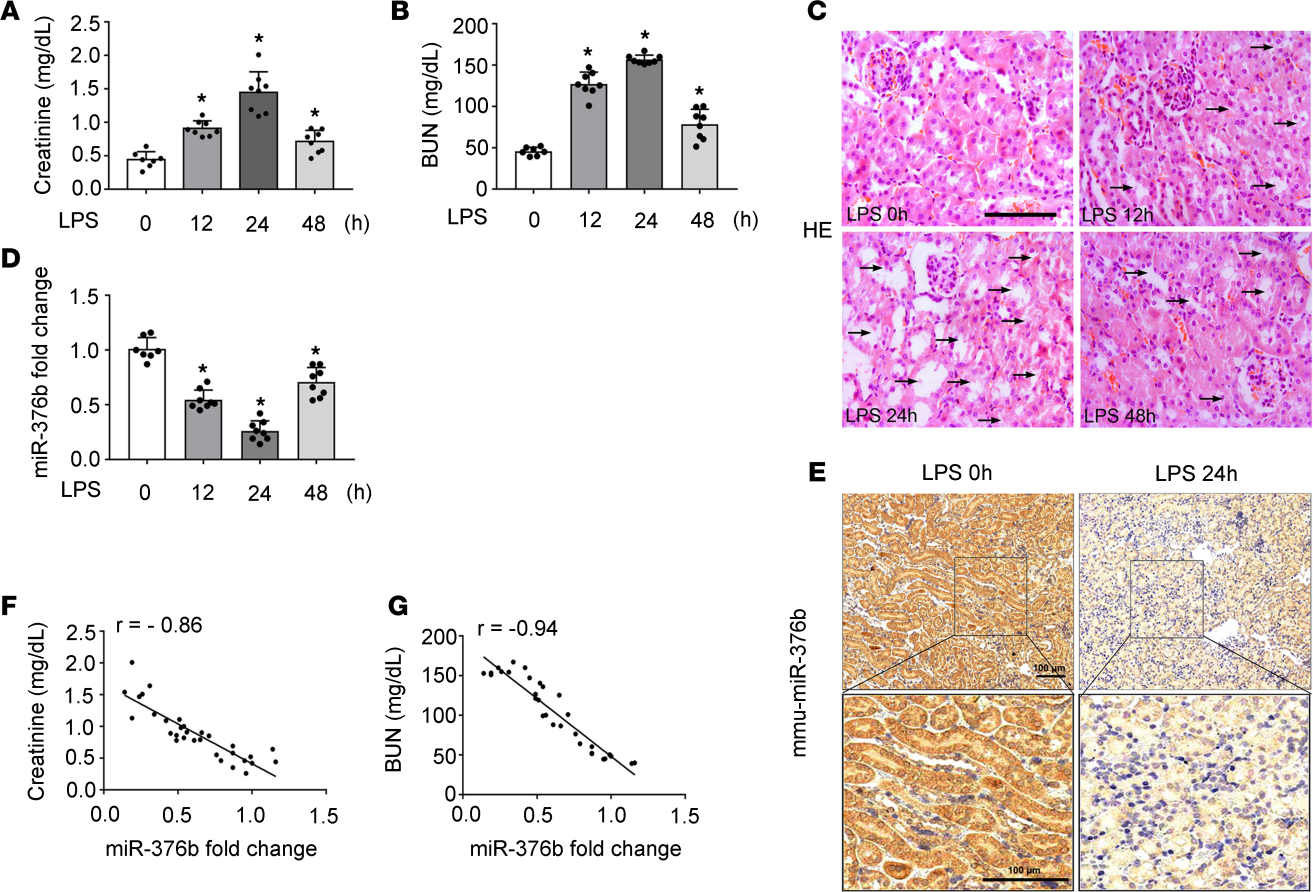
miR-376b is downregulated in renal tubular cells during LPS-induced endotoxic AKI. Male C57BL/6 mice were injected with one dose of LPS (10 mg/kg) and samples were collected at indicated time points. Control mice (LPS 0h) were injected with normal saline. All quantitative data are expressed as mean ± SD (*n* = 7 or *n* = 8, 2-tailed Student’s *t* test), *P < 0.05 vs. control. (**A**) Time-dependent increase of serum creatinine in LPS-treated mice. (**B**) Time-dependent increase of BUN in LPS-treated mice. (**C**) Representative images of H&E staining. Scale bar: 50 μm. (**D**) qPCR analysis showing miR-376b decrease in kidneys in LPS-treated mice. The level of miR-376b was normalized to the level of sno202 (internal loading control) of the same samples to determine the ratio with the ratio of control mice arbitrarily set as 1. (**E**) In situ hybridization showing miR-376b decrease in kidney tissues after LPS treatment. Images on the bottom row show high-magnification views of the boxed areas in the top row. Scale bar: 100 μm. (**F**) Correlation analysis of miR-376b expression in renal tissue with serum creatinine in LPS-treated mice (r = –0.86, Spearman’s correlation test). (**G**) Correlation analysis of miR-376b expression in renal tissue with BUN in LPS-treated mice (r = –0.94, Spearman’s correlation test).

To identify specific miRNAs involved in the pathogenesis of septic AKI, we initially tested the mouse model of LPS treatment. As shown in Figure 1, A and B, the levels of serum creatinine and blood urea nitrogen (BUN) in the mice increased at 12 hours after 10 mg/Kg LPS treatment and peaked at 24 hours after LPS injection. H&E staining showed kidney injury at the histology level (Figure 1C), especially at 24 hours after LPS injection. We collected kidney cortical tissues from the mice at 24 hours after LPS treatment for microarray analysis of miRNA expression. The analysis revealed a series of miRNAs with altered expression, including miR-376b. By TaqMan real-time PCR, we further verified the decrease of miR-376b in the kidneys of the LPS-treated mice as compared with the control mice (Figure 1D). Interestingly, miR-376b expression started to decrease at 12 hours after LPS treatment, reached the lowest level at 24 hours, and then recovered to some degree at 48 hours after treatment. In situ hybridization analysis confirmed LPS-induced miR-376b downregulation mainly in the renal tubules in the cortex and outer medulla (Figure 1E). Spearman’s test further established a negative correlation between kidney miR-376b and serum creatinine or BUN in LPS-treated mice (Figure 1, F and G). LPS also induced a decrease of miR-376b in cultured Boston University mouse proximal tubular cell line (BUMPT) cells, a mouse proximal tubular cell line (Supplemental Figure 1; supplemental material available online with this article; https://doi.org/10.1172/jci.insight.142272DS1).

### Decrease of miR-376b in renal tubules of CLP-induced septic mice.

**Figure 2:**
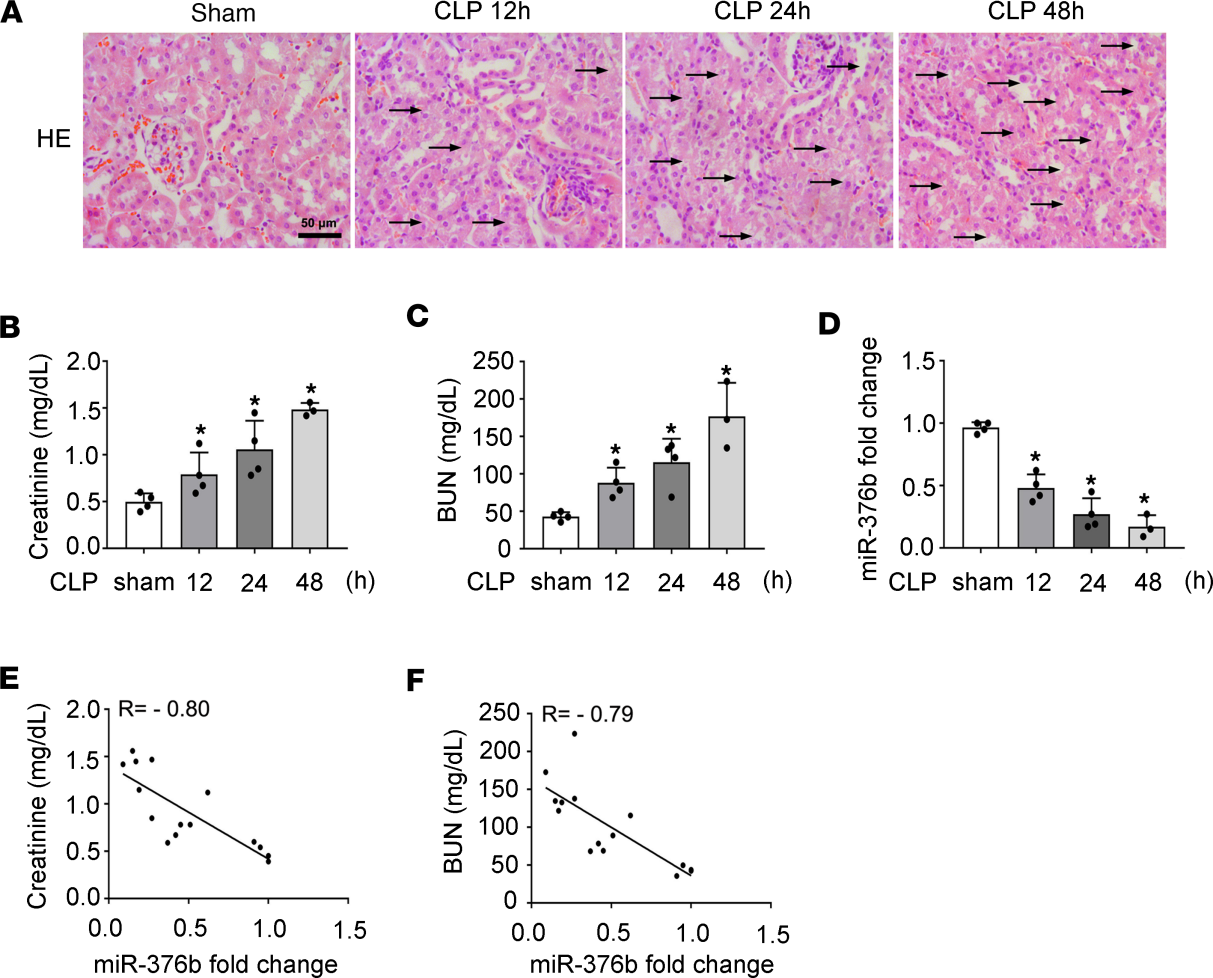
Decrease of miR-376b in renal tubules in CLP-induced septic AKI. Male C57BL/6 mice were subjected CLP or sham operation, and samples were collected at indicated time points. All quantitative data are expressed as mean ± SD (*n* = 3 or *n* = 4, 2-tailed Student’s *t* test), *P < 0.05 vs. sham. (**A**) Representative images of HE staining of kidney tissues. Scale bar: 50 μm. (**B**) Increase of serum creatinine in CLP-induced septic AKI in mice. (**C**) Increase of BUN in CLP-induced septic AKI in mice. (**D**) miR-376b decrease in mouse kidneys after CLP-induced sepsis shown by qPCR analysis. The levels of miR-376b were normalized to the levels of sno202 of the same samples to determine the ratios. The ratios of sham were arbitrarily set as 1. (**E**) Correlation analysis of the levels of miR-376b in renal tissue with serum creatinine in CLP-treated mice (r = –0.80, Spearman’s correlation test). (**F**) Correlation analysis of miR-376b in renal tissue with BUN in CLP-treated mice (r = –0.79, Spearman’s correlation test).

While LPS treatment induces endotoxic AKI, CLP may be a more clinically relevant septic model (29). Therefore, we further evaluated miR-376b in kidney tissues of CLP-induced mice. In histological and functional analyses, CLP induced severe renal dysfunction and tissue damage at 48 hours (Figure 2, A–C), which was accompanied by a dramatic decrease of miR-376b in renal tissues (Figure 2D). In addition, the level of renal miR-376b negatively correlated with the levels of serum creatinine and BUN in the CLP experiment (Figure 2E, r = –0.80, Spearman’s correlation test, and Figure 2F, r = –0.79, Spearman’s correlation test).

### NF-κB mediates the downregulation of miR-376b in septic AKI.

**Figure 3:**
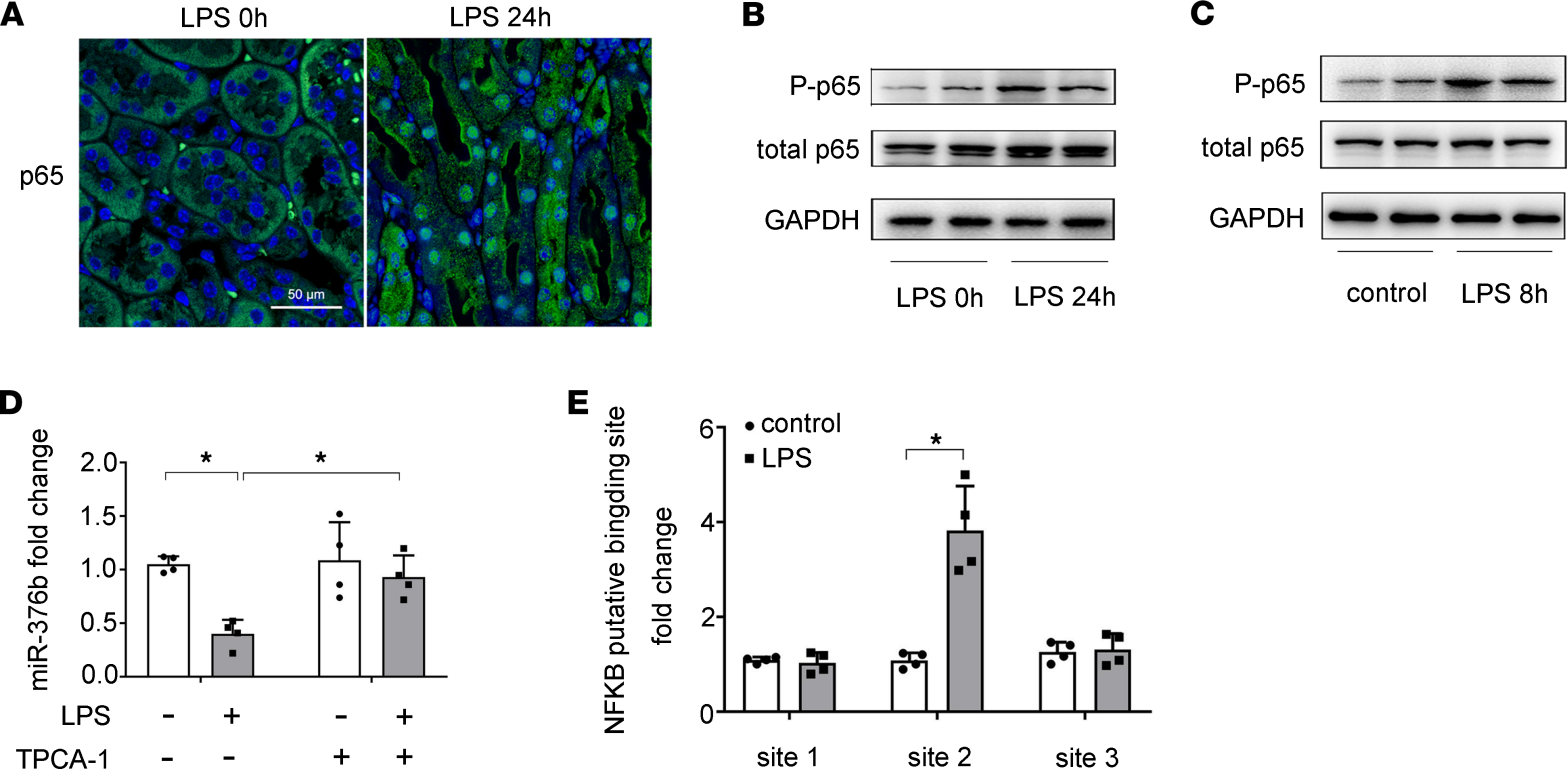
NF-κB mediates the downregulation of miR-376b in LPS-induced AKI. (**A**) Immunofluorescence showing nuclear translocation of p65/NF-κB in renal tubule cells in LPS-treated mice. Images were collected by laser scanned confocal microscopy. Scale bar: 50 μm. (**B**) Immunoblots showing increases of p-p65 and p65 in kidney tissues of LPS-treated mice. GAPDH was used as internal control. (**C**) Immunoblots showing LPS-induced p-p65 and p65 in BUMPT cells. GAPDH was used as internal control. (**D**) qPCR analysis showing that LPS-induced decrease of miR-376b is prevented by TPCA-1. BUMPT cells were treated with 100 μM TPCA-1 and then treated with LPS. Total RNA samples were extracted after LPS treatment to quantify miR-376b by qPCR. The levels of miR-376b were normalized to the levels of sno202 of the same samples to determine the ratios. Data are expressed as mean ± SD (*n* = 4, 1-way ANOVA with Tukey’s multiple comparisons test), *P < 0.05. (**E**) ChIP analysis of NF-κB binding in miR-376b promoter. BUMPT cells were treated with or without LPS for 8 hours to collect the chromatin for immunoprecipitation with a specific anti–NF-κB antibody. The immunoprecipitated samples were subjected to qPCR analysis of miR-376b promoter sequence. Quantitative data are expressed as mean ± SD (*n* = 4, 2-tailed Student’s *t* test), *P < 0.05.

NF-κB contributes critically to intrarenal inflammation by transcriptionally regulating downstream genes, especially proinflammatory cytokines (17, 18). We hypothesized that NF-κB might have a role in miR-376 downregulation observed in septic AKI. By immunofluorescence, we detected an obvious increase in the number of renal tubular cells with positive nuclear staining of p65, a member of the NF-κB family, at 24 hours after LPS exposure (Figure 3A). Immunoblot analysis detected increases in both total p65 and phosphorylated/activated p65 (p-p65) in kidneys of LPS-treated mice (Figure 3B). In vitro, LPS induced a marked increase of p-p65 in BUMPT cells, although the induction of total p65 was not obvious (Figure 3C). These results verified LPS-induced NF-κB activation in renal tubular cells. To determine the involvement of NF-κB in LPS-induced downregulation of miR-376b, we tested the effect of TPCA-1, a specific IKK inhibitor that prevents NF-κB activation by blocking IκB phosphorylation and dissociation from NF-κB (30). As shown in Figure 3D, TPCA-1 prevented an miR-376b decrease during LPS treatment of BUMPT cells. Bioinformatics analysis using the JASPAR Database (http://jaspar.genereg.net/) identified 3 potential NF-κB binding sites at the gene promoter of miR-376b, designated as sites 1, 2, and 3 (Table 1). ChIP assay demonstrated that LPS specifically increased the binding of p65/NF-κB to the site 2 sequence of the gene promoter of miR-376b (Figure 3E). These results suggest that NF-κB may mediate miR-376b downregulation in septic AKI by directly regulating gene transcription.

### miR-376b promotes apoptosis and inflammatory response during LPS treatment of BUMPT cells.

**Figure 4:**
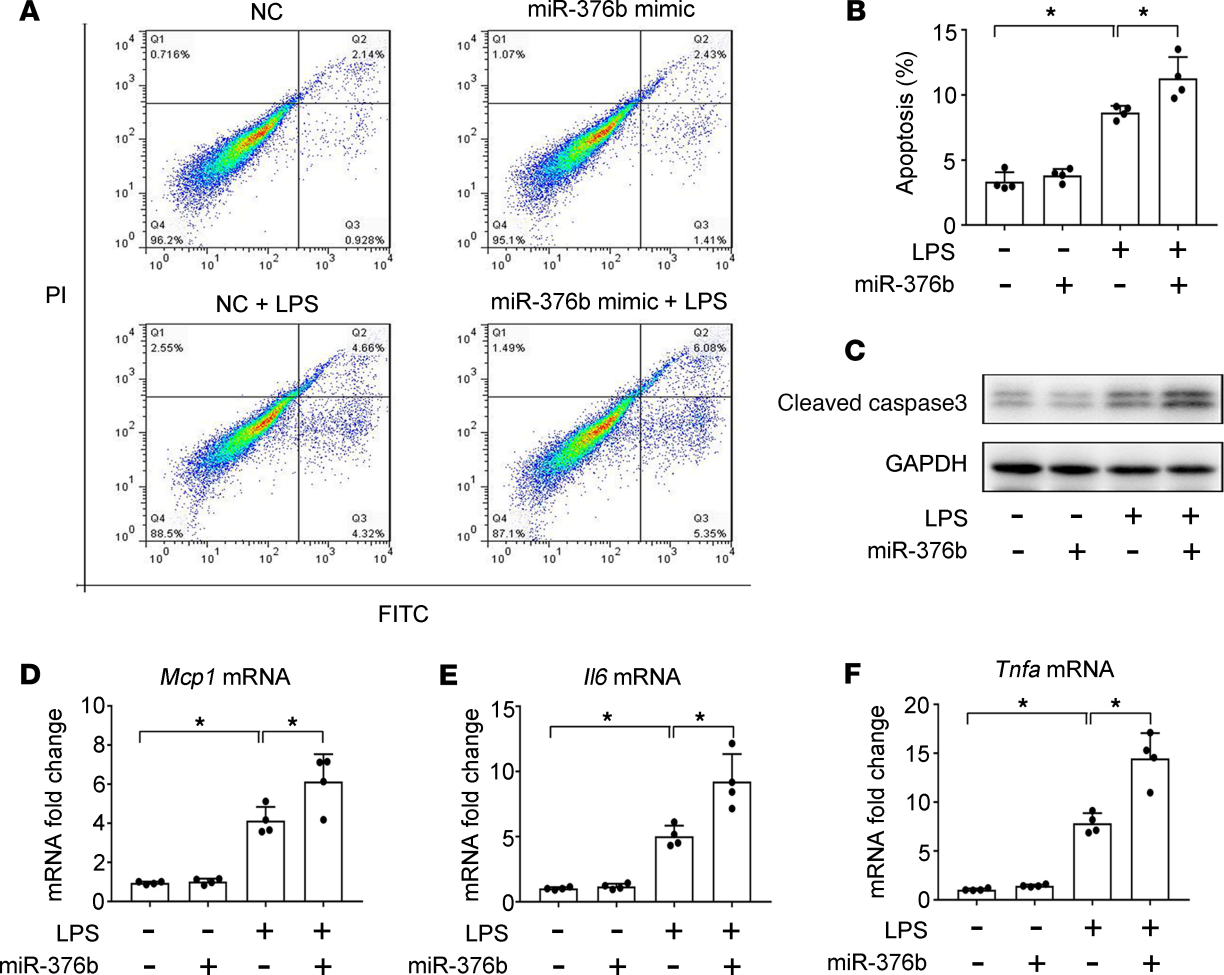
miR-376b mimic promotes cell death and inflammatory response in LPS-treated BUMPT cells. BUMPT cells were transfected with 200 nM miR-376b mimics or NC sequences and then treated with 10 μg/mL LPS for 8 hours. (**A** and **B**) Flow cytometry analysis showing that miR376 mimic increases cell death during LPS treatment of BUMPT cells. After treatment, the cells were stained with annexin V and propidium iodide (PI) for flow cytometry. Quantitative data are expressed as mean ± SD (*n* = 4, 1-way ANOVA with Tukey’s multiple comparisons test), *P < 0.05. (**C**) Immunoblot analysis of active/cleaved caspase-3. GAPDH was used as a loading control. (**D**–**F**) qPCR analysis showing that miR376 mimic increases the expression of *Mcp1*, *Il6*, and *Tnfa* during LPS treatment of BUMPT cells. Data are expressed as mean ± SD (*n* = 4, 1-way ANOVA with Tukey’s multiple comparisons test), *P < 0.05.

To elucidate the role of miR-376b in septic AKI, we initially evaluated the effect of transfection of miR-376b mimics on LPS-induced cell death in BUMPT cells. In the control group, flow cytometry analysis detected approximately 3% dead cells that were positive for annexin V or propidium iodide (PI) staining, which was increased to approximately 9% by LPS treatment. The miR-376b mimic further increased cell death to approximately 12% (Figure 4, A and B). Consistently, immunoblot analysis showed more cleaved/active caspase-3 in the cells exposed to LPS plus miR-376b mimic versus LPS alone (Figure 4C). We further measured the production of inflammatory cytokines in BUMPT cells by quantitative real-time PCR (qPCR) (Figure 4, D–F). LPS induced the production of *Mcp1*, *Il6*, and *Tnfa* mRNA, which was further enhanced by the miR-376b mimic. Of note, miR-376b alone showed minimal effects on cell death and cytokine production in BUMPT cells (Figure 4, A–F). Taken together, these results indicate a prodeath and proinflammatory function of miR-376b during LPS treatment of renal tubular cells, suggesting that downregulation of miR-376b in septic AKI is a protective response.

### miR-376b exaggerates LPS-induced AKI in mice.

**Figure 5:**
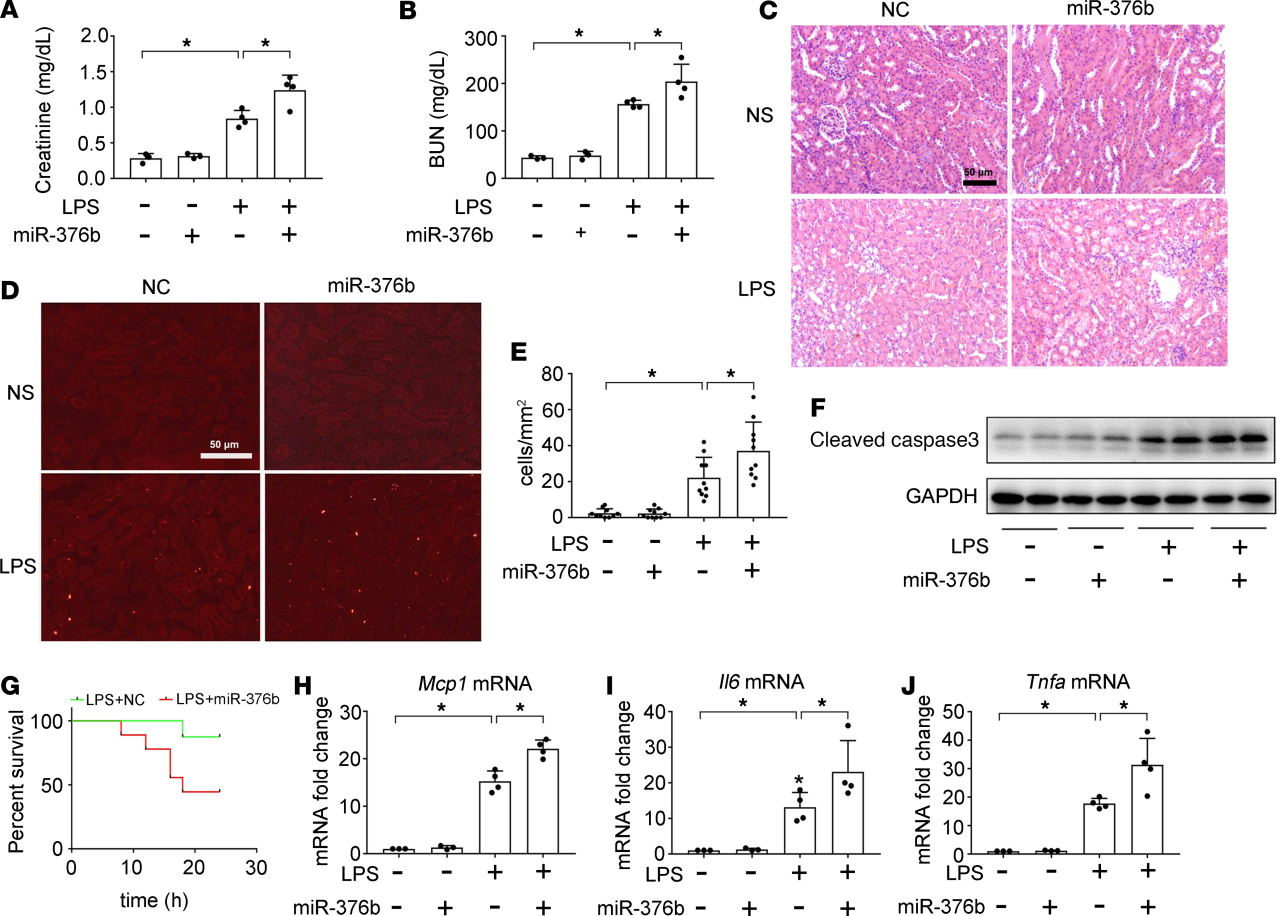
miR-376b mimic worsens LPS-induced septic AKI in mice. miR-376b mimic (7mg/kg) or NC oligonucleotides were injected into male C57BL/6 mice through tail vein. One day later, the mice were injected intraperitoneally with 10 mg/kg LPS. Control mice were injected with normal saline (NS). Kidney tissues were collected at 24 hours after LPS injection. (**A**) Serum creatinine. (**B**) BUN. (**C**) Representative images of H&E staining. Scale bar: 50 μm. (**D**) Representative images of TUNEL staining. Scale bar: 50 μm. (**E**) Quantification of TUNEL-positive cells. (**F**) Immunoblot analysis of active caspase-3 in septic AKI kidneys. GAPDH was used as loading control. (**G**) Kaplan-Meier curves showing miR-376b mimic worsens animal death after LPS exposure. (**H**–**J**) qPCR analysis of *Mcp1*, *Il6*, and *Tnfa* mRNA. All quantitative data were expressed as mean ± SD (*n* = 3 or *n* = 4 in **A**, **B**, and **H**–**J** and *n* = 10 in **E**, 1-way ANOVA with Tukey’s multiple comparisons test),*P < 0.05.

We further evaluated the effects of miR-376b mimics on septic AKI in vivo. First, we confirmed that the in vivo delivery of the miR-376b mimic resulted in a sustained increase of miR-376b in kidney tissues (Supplemental Figure 2). In control mice, the miR-376b mimic did not cause functional or structural damages in kidneys, but it significantly increased the levels of serum creatinine and BUN and aggravated kidney tissue damage in LPS-treated mice (Figure 5, A–C). TUNEL assay showed that miR-376b mimics increased renal tubular cell death during LPS treatment (Figure 5, D and E). Immunoblot analysis also detected more cleaved/active caspase-3 in the kidney tissues of LPS+miR-376b–treated mice than in mice treated with LPS only (Figure 5F). Moreover, animal death occurred earlier in the LPS+miR-376b–treated group than in the LPS only group with negative control (NC) oligonucleotides. The LPS+miR-376b–treated group also had a notably higher mortality rate, as shown by Kaplan-Meier curves (Figure 5G). The miR-376b mimic also enhanced the production of proinflammatory cytokines in LPS-treated mice (Figure 5, H–J). These findings further support a pathogenic role of miR-376b in septic AKI.

### Inhibition of miR-376b attenuates LPS-induced septic AKI.

**Figure 6:**
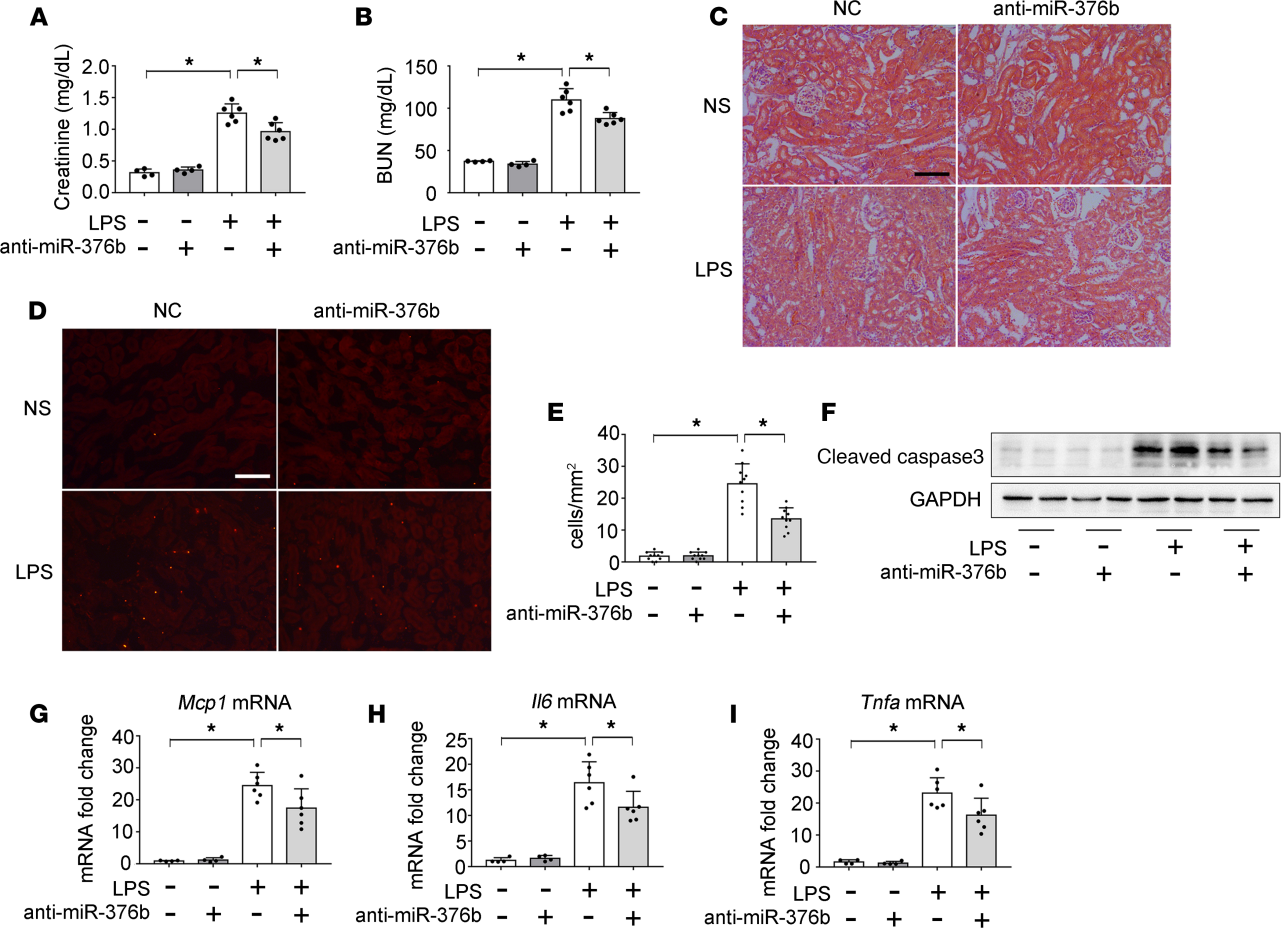
Inhibition of miR-376b attenuates LPS-induced septic AKI in mice. C57BL/6 male mice were given 20 mg/kg anti–miR-376b or scrambled sequence oligonucleotides (negative control [NC]) and then injected with 10 mg/kg LPS or normal saline (NS) as control. Kidney tissues were collected at 24 hours after LPS injection. (**A**) Serum creatinine. (**B**) BUN. (**C**) Representative images of H&E staining. Scale bar: 100 μm. (**D**) Representative images of TUNEL staining. Scale bar: 100 μm. (**E**) Quantification of TUNEL-positive cells. (**F**) Immunoblot analysis of active caspase-3 in kidney cortical tissues. GAPDH was used as loading control. (**G**–**I**) qPCR analysis of *Mcp1*, *Il6*, and *Tnfa* mRNA. All quantitative data were expressed as mean ± SD (*n* = 4 or *n* = 6 in **A**, **B**, and **G**–**I** and *n* = 10 in **E**, 1-way ANOVA with Tukey’s multiple comparisons test), *P < 0.05.

To further determine the role of miR-376b in septic AKI, we examined the effect of anti–miR-376b. Locked nucleic acid–modified (LNA-modified) anti–miR-376b or scrambled sequence oligonucleotides (NC) were administered to mice. Anti–miR-376b caused neither renal functional nor structural changes in control mice, but it significantly reduced the levels of serum creatinine and BUN and attenuated kidney tissue damage in LPS-induced septic mice (Figure 6, A–C). Consistently, anti–miR-376b reduced renal apoptosis in this model, as shown by TUNEL staining (Figure 6, D and E) and active caspase-3 immunoblotting (Figure 6F). Moreover, anti–miR-376b inhibited the production of proinflammatory cytokines in LPS-treated mice (Figure 6, G–I). These results further support the conclusion that miR-376b plays a proinflammatory and proinjury role in septic AKI.

### NFKBIZ is a downstream target of miR-376b in septic AKI.

**Figure 7:**
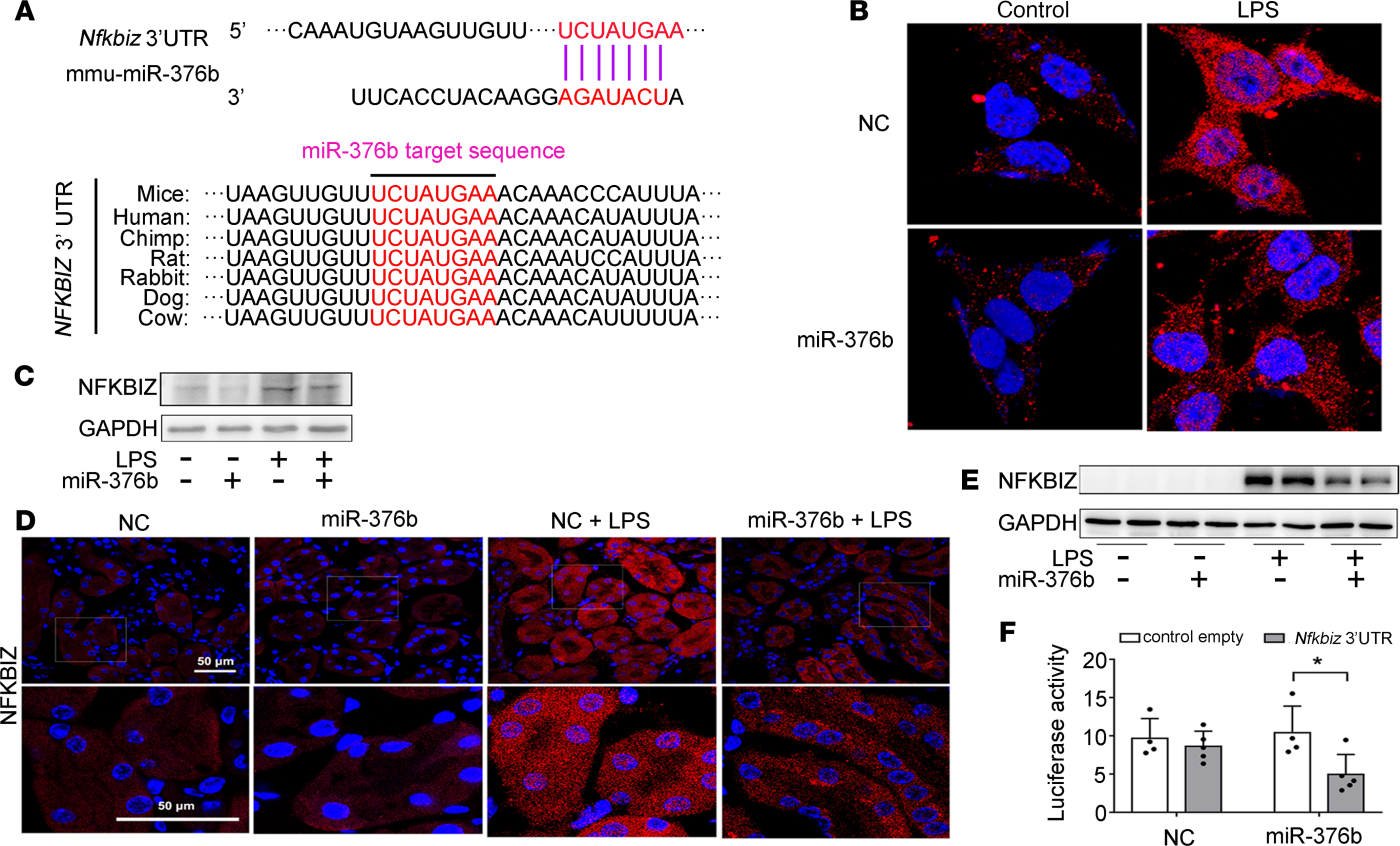
miR-376b targets NFKBIZ in LPS induced septic AKI. (**A**) Conserved miR-376b target sequence in the 3′-UTR of *NFKBIZ* mRNA. (**B**) Immunofluorescence showing the repressive effect of miR-376b on NFKBIZ expression. BUMPT cells were transfected with 200 nM miR-376b mimic or NC for 24 hours and then treated with or without 10 μg/mL LPS for 8 hours. (**C**) Immunoblot analysis showing the repressive effect of miR-376b on NFKBIZ expression. BUMPT cells were treated as in **B** to collect lysate for immunoblot analysis. GAPDH was used as internal control in immunoblot analysis. (**D**) Immunofluorescence showing the repressive effect of miR-376b on NFKBIZ expression. miR-376b mimic (7 mg/kg) or NC oligonucleotides were delivered to male C57BL/6 mice through tail vein injection 1 day before LPS injection. The mice were then injected intraperitoneally with 10 mg/kg LPS, while control mice were injected with normal saline. Kidney tissues were collected at 24 hours after LPS injection. Scale bar: 100 μm. (**E**) Immunoblot analysis showing the repressive effect of miR-376b on NFKBIZ expression. Mice were subjected to the same treatment as in **D** to collect kidney tissues for immunoblot analysis. GAPDH was used as internal control. (**F**) MicroRNA target reporter assay of *Nfkbiz* 3′-UTR. The putative miR-376b target sequence of the *Nfkbizc* 3′-UTR was cloned into the pMIR-REPORT vector. This and empty vector were transfected with miR-376b mimic or NC oligonucleotides to analyze luciferase activity. miR-376b mimic specifically reduced luciferase expression by pMIR-REPORT *Nfkbiz* 3′-UTR. Quantitative data are expressed as mean ± SD (*n* = 4 or *n* = 5, 2-tailed Student’s *t* test), *P < 0.05.

To understand the mechanism whereby miR-376b contributes to septic AKI, we investigated it downstream of target gene(s). Bioinformatic analysis with online databases (miRBase, http://www.mirbase.org/, and TargetScan, http://www.targetscan.org/vert_72/) identified a putative miR-376b targeting sequence in the 3′-UTR of mouse *Nfkbiz* mRNA, which is highly conserved across animal species (Figure 7A). To determine whether *Nfkbiz* is indeed a target of miR-376b, we first evaluated the effect of miR-376b on the expression of NFKBIZ. In immunofluorescence staining, LPS treatment induced NFKBIZ in BUMPT cells and this induction was markedly suppressed by miR-376b mimic (Figure 7B). Consistently, immunoblot analysis demonstrated that transfection of miR-376b mimic reduced the expression of NFKBIZ in BUMPT cell exposed to LPS (Figure 7C and Supplemental Figure 3). Notably, LPS also induced NFKBIZ expression in renal tubule cells in mice, which was suppressed by miR-376b mimic (Figure 7D). The in vivo suppressive effect of miR-376 on NFKBIZ was further verified by immunoblot analysis (Figure 7E). To determine if NFKBIZ is a direct target of miR-376b, we performed luciferase microRNA target reporter assay. The *Nfkbiz* 3′-UTR sequence was cloned into the microRNA Luciferase reporter construct, which was then cotransfected with miR-376b mimic or NC oligonucleotides into HEK293 cells. As shown in Figure 7F, cotransfection of miR-376b mimic (but not NC oligonucleotides) suppressed luciferase expression from the reporter construct with *Nfkbiz* 3′-UTR. Collectively, these results indicate that miR-376b may directly target *Nfkbiz* mRNA to repress its expression in septic AKI.

### Knockdown of Nfkbiz enhances LPS-induced apoptosis and inflammatory response in BUMPT cells.

**Figure 8:**
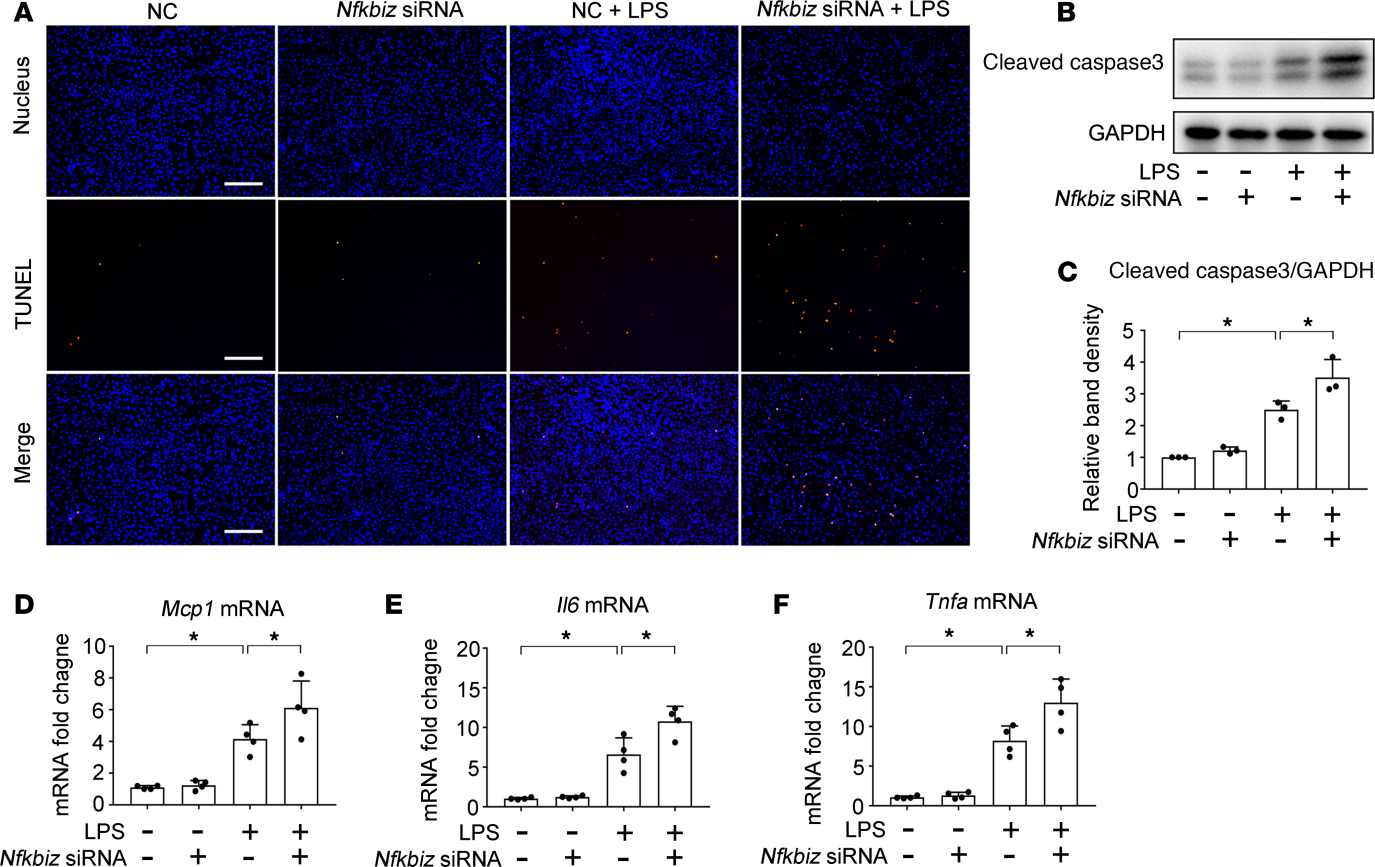
Silencing *Nfkbiz* enhances LPS-induced apoptosis and inflammatory response in BUMPT cells. BUMPT cells were transfected with 40 nM *Nfkbiz* siRNA or NC oligonucleotides and then treated with 10 μg/mL LPS for 8 hours. (**A**) Representative images of TUNEL staining. Scale bar: 200 μm. (**B**) Immunoblot analysis of active/cleaved caspase-3. GAPDH was used as a loading control. (**C**) quantification of active caspase-3. Protein bands in immunoblots were analyzed by densitometry to calculate the signal ratio of active caspase-3/GAPDH with the ratio in control cells were arbitrarily set as 1. Data are expressed as mean ± SD (*n* = 3, 1-way ANOVA with Tukey’s multiple comparisons test), *P < 0.05. (**D**–**F**) qPCR analysis for *Mcp1*, *Il6*, and *Tnfa*. Quantitative data are expressed as mean ± SD (*n* = 4, 1-way ANOVA with Tukey’s multiple comparisons test),*P < 0.05.

NFKBIZ is an atypical member of the IκB family, which may inhibit NF-κB and the associated inflammatory response(18, 31). To determine the role of NFKBIZ in septic AKI, we examined the effects of *Nfkbiz* knockdown on apoptosis and cytokines production in BUMPT cells during LPS treatment. Transfection of *Nfkbiz* siRNA dramatically reduced NFKBIZ expression (Supplemental Figure 4, A and B). Importantly, LPS induced more cell death in *Nfkbiz*-knockdown cells than in the cells transfected with control sequences (Figure 8A). *Nfkbiz*-knockdown cells also had higher levels of caspase-3 activation upon LPS treatment, indicative of more apoptosis (Figure 8, B and C). LPS induced the production of proinflammatory cytokines, such as *Mcp1*, *Il6* and *Tnfa*, and this inductive response was more pronounced in *Nfkbiz*-knockdown cells (Figure 8, D–F). Thus, NFKBIZ may play a protective role against tubular cell death and inflammation in septic AKI.

### Urinary miR-376b is a potential biomarker for septic AKI.

**Figure 9:**
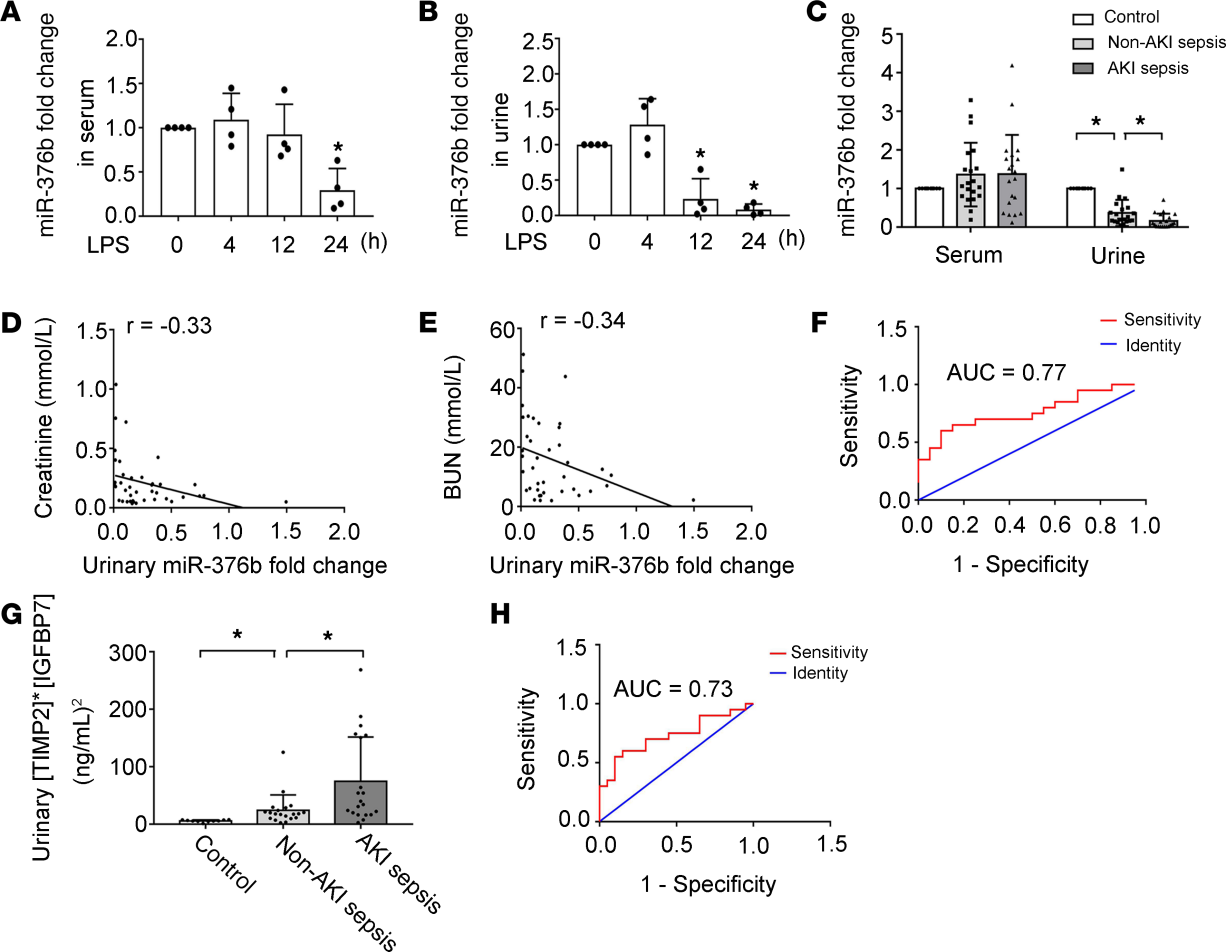
Evaluation of urinary miR-376b as a biomarker for septic AKI. (**A** and **B**) Male C57BL/6 mice were injected with 10 mg/kg LPS to collect serum and urine samples at indicated time points to measure miR-376b. The values were normalized with that of control mice (LPS time 0) which was arbitrarily set as 1. Serum miR-376b showed a significant decrease at 24 hours after LPS exposure (**A**), whereas urinary miR-376b showed a significant decrease at 12 and 24 hours (**B**). All values in **A** and **B** are presented as mean ± SD (*n* = 4, 2-tailed Student’s *t* test), *P < 0.05 vs. control mice. (**C**) Serum and urinary miR-376b in healthy subjects (control) and patients with sepsis with or without AKI. The values were normalized with that of serum or urinary miR-376b of healthy subjects (control), which was arbitrarily set as 1. (**D**) Correlation analysis of urinary miR-376b with serum creatinine in patients with sepsis (r = –0.33 Spearman’s correlation test). (**E**) Correlation analysis of urinary miR-376b with BUN in patients with sepsis (r = –0.34, Spearman’s correlation test). (**F**) ROC curves for the detection of septic AKI in patients with sepsis using urinary miR-376b. (**G**) Urinary [TIMP2]*[IGFBP7] in healthy subjects and patients with sepsis with or without AKI. (**H**) ROC curves for the detection of AKI in patients with sepsis using urinary [TIMP2]*[IGFBP7]. Values in **C** and **G** are presented as mean ± SD (*n* = 10 or *n* = 20, 1-way ANOVA with Tukey’s multiple comparisons test), *P < 0.05.

As described above, the levels of miR-376b in renal tissues decreased dramatically in the mouse models of LPS- and CLP-induced septic AKI, and this decrease showed a significant correlation with the increases of serum creatinine and BUN (Figure 1, F and G, and Figure 2, E and F), suggesting that miR-376b is a potential biomarker for septic AKI. We therefore analyzed serum and urinary miR-376b in these mouse models. In serum, miR-376b did not decrease at either 4 or 12 hours after LPS treatment, and at 24 hours miR-376b decreased to approximately 30% of the control level (Figure 9A). In contrast, urinary miR-376b decreased to less than 20% of control at 12 hours after LPS treatment and further decreased to less than 10% at 24 hours (Figure 9B). We further compared the levels of serum and urinary miR-376b in patients with sepsis with or without AKI. Samples were collected from 10 healthy people and 40 patients with sepsis, 20 of whom had AKI while the others did not. The clinical information for these participants is presented in Supplemental Table 1. As expected, the patients with sepsis and AKI had significantly higher levels of serum creatinine (345.20 vs. 76.71 μmol/L) and BUN (26.24 vs. 5.72 mmol/L) than did the patients with sepsis without AKI. Otherwise, these 2 groups of patients were similar in age, sex, serum albumin, hemoglobin, C-reactive protein (CRP), calcitonin, and neutrophil count. In serum, miR-376b did not show significant differences between healthy control and patients with sepsis, regardless of AKI. However, the healthy subjects had higher urinary miR-376b than the patients with sepsis. Notably, in patients with sepsis, those without AKI had significantly higher urinary miR-376b than those with AKI (Figure 9C). Moreover, Spearman’s test demonstrated a negative correlation of urinary miR-376b with serum creatinine and BUN in patients with sepsis (Figure 9, D and E). To evaluate the diagnostic ability of urinary miR-376b for septic AKI, we conducted the receiver operating characteristic (ROC) analysis. As show in Figure 9F and Table 2, the AUC was 0.77 for urinary miR-376b and the optimal cut-point was 0.1428-fold for AKI diagnosis in the patients with sepsis. Based on the optimal cut-point, urinary miR-376b achieved a sensitivity of 65% and a specificity of 85% for the detection of AKI in patients with sepsis (Table 2). We further examined the diagnostic efficiency of urinary miR-376b by binary logistic regression analysis, which showed a 47.6-fold increase of the risk of AKI in the patients with sepsis for every 1-fold decrease in urinary miR-376b (Table 3). NephroCheck, based on the arithmetic result of urinary tissue inhibitor of metalloproteinase 2 (TIMP2) and insulin-like growth factor binding protein 7 (IGFBP7) ([TIMP2]*[IGFBP7]), is the only biomarker test recently approved by the FDA for AKI detection in patients (22, 32). We compared the efficacies of urinary miR-376b and urinary [TIMP2]*[IGFBP7] in the diagnosis of patients with AKI and sepsis. The mean values of urinary [TIMP2]*[IGFBP7] were significantly higher in the patients with sepsis and AKI than in those without AKI (Figure 9G). The AUC for [TIMP2]*[IGFBP7] was 0.73, which was slightly lower than the AUC of 0.77 for urinary miR-376b (Figure 9H). Urinary [TIMP2]*[IGFBP7] showed a sensitivity of 60% and a specificity of 85% for AKI diagnosis in the patients with sepsis, while urinary miR-376b had a sensitivity of 65% and a specificity of 85% (Table 2). Thus, urinary miR-376b had a slightly higher sensitivity and a comparable specificity for detecting AKI in patients with sepsis.

## Discussion

**Figure 10:**
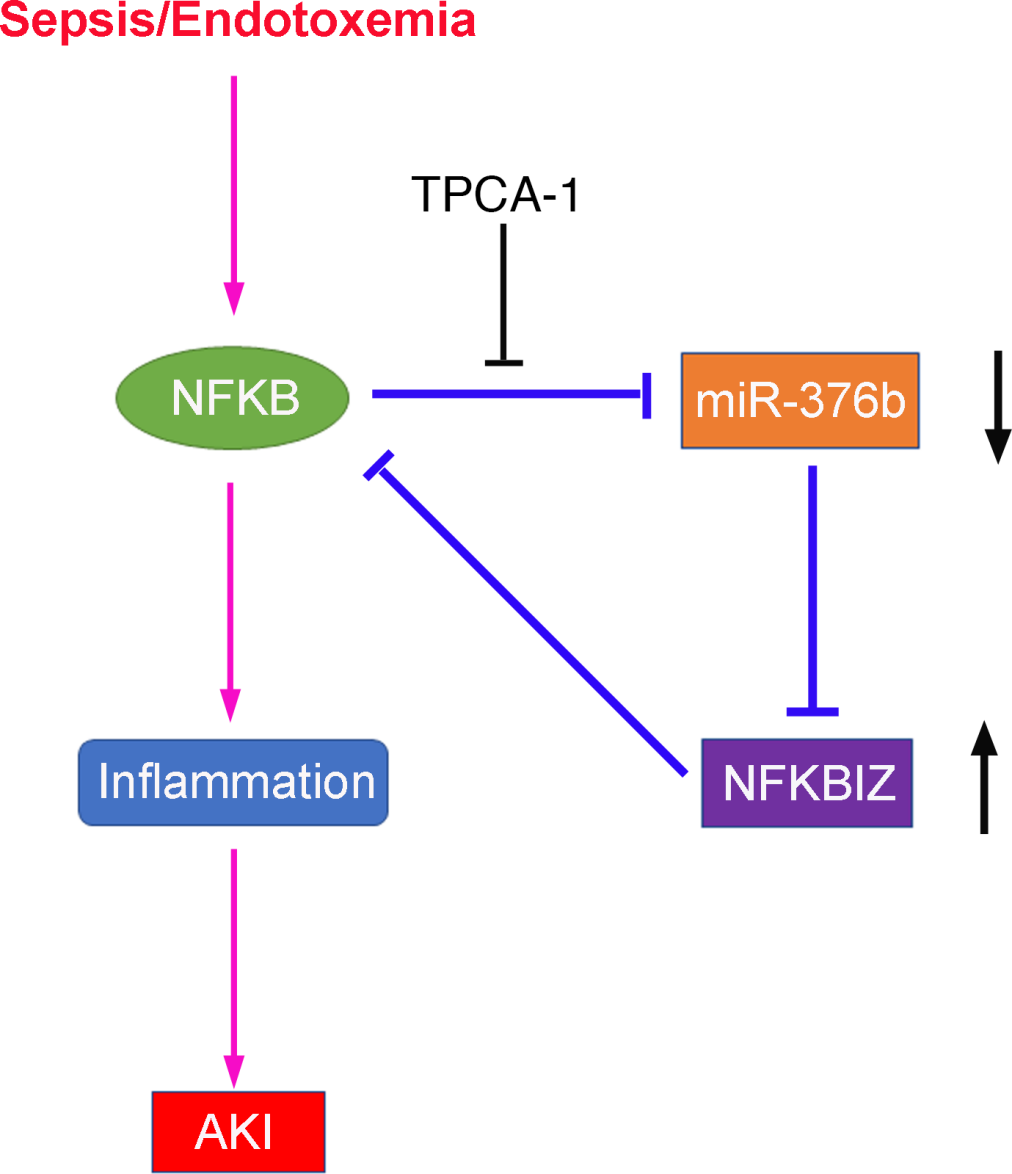
NF-κB/miR-376b/NFKBIZ negative feedback mechanism in septic AKI. In sepsis, NF-κB is activated to induce the production of proinflammatory cytokines for inflammation and AKI. However, upon activation, NF-κB may also inhibit the expression of miR-376b in renal tubular cells, leading to the induction of NFKBIZ, which provides a negative feedback mechanism to suppress NF-κB.

Clinically, nearly half of AKI cases are caused by sepsis, but how sepsis induces kidney injury and functional loss remains elusive. In this study, we report the following major findings: (a) miR-376b is dramatically downregulated in renal tubular cells in both in vitro and in vivo models of septic AKI, and it did not change in ischemic or cisplatin-induced AKI (Supplemental Figure 5); (b) the downregulation of miR-376b is mediated by NF-κB; (c) functionally, miR-376b increases the production of proinflammatory cytokines and tubular cell death, indicating that miR-376b downregulation in septic AKI is an adaptive or protective mechanism; (d) mechanistically, miR-376b may directly target and repress NFKBIZ to promote NF-κB–associated inflammation and inflammatory cell death in septic AKI; and (e) decrease of urinary miR-376b may be a useful biomarker for the diagnosis or identification of AKI in patients with sepsis. Together, these findings unveil a potentially novel mechanism of NF-κB regulation involving the negative feedback of miR-376b and NFKBIZ. Essentially, NF-κB is activated in sepsis to suppress the expression of miR-376b, leading to the induction of its target gene *NFKBIZ*. NFKBIZ then antagonizes NF-κB to reduce the expression of proinflammatory genes and tubular cell death (Figure 10).

miRNAs have been implicated in the pathogenesis of AKI (23), but specific miRNAs involved in septic AKI remain largely unknown. In the present study, we have demonstrated compelling evidence to support an injurious role of miR-376b in septic AKI. In vitro, LPS induced a significant decrease in BUMPT cells. In both mouse models induced by LPS and CLP, miR-376b was downregulated in renal tubules, and this downregulation correlated well with the development of AKI (Figure 1). Notably, patients with sepsis and AKI had significantly less miR-376b in urine than patients with sepsis without AKI (Figure 9). Moreover, supplementation of miR-376b mimics led to increased cell death and inflammatory response in BUMPT cells and an exaggerated AKI following LPS treatment in mice (Figures 4 and 5), whereas inhibition of miR-376b had kidney protective effects in this model (Figure 6). These results indicate an injurious role of miR-376b in septic AKI. Accordingly, miR-376b downregulation in septic AKI is an adaptive or protective response in this disease condition.

The development of AKI involves multiple cell types, including renal resident cells and circulating leukocytes (1, 33). Our in situ hybridization analysis localized miR-376b expression mainly in renal tubule cells of the renal cortex, and its downregulation also occurred in these cells following sepsis. Moreover, there was a significant decrease of miR-376b in urine in septic AKI mice and in patients with sepsis and AKI, suggesting that renal tubular cells are the major source of urinary miR-376b.

Regarding the mechanism of miR-376b downregulation in septic AKI, we have suggested a role of NF-κB (Figure 3). NF-κB was activated in BUMPT cells exposed to LPS and in renal tubules in mouse models of septic AKI, as indicated by p65 phosphorylation and nuclear translocation. Moreover, inhibition of NF-κB with TPCA-1 prevented the decrease of miR-376b in BUMPT cells during LPS treatment. In addition, ChIP assay demonstrated that NF-κB (p65) bound to a specific site on the promoter of the miR-376b gene, and the binding was enhanced by LPS treatment (Figure 3). Together, these results indicate that NF-κB suppressed miR-376b expression in septic AKI by directly binding to its gene promoter. This observation is somewhat surprising, because NF-κB is generally known to activate target gene expression. Nonetheless, NF-κB has been reported to suppress gene expression in a rat model of myocardial ischemia (34). How NF-κB represses the transcription of target genes awaits future investigation.

How does miR-376b contribute to septic AKI? To address this, our in vitro and in vivo studies have identified NFKBIZ as a direct target of miR-376b (Figure 7). NFKBIZ belongs to the IκB family of NF-κB inhibitory proteins (35, 36). Similar to other IκB proteins, NFKBIZ contains carboxyl terminal ankyrin-repeat domains that are essential for its binding to NF-κB (37). Unlike the classical IκB family members, NFKBIZ does not inhibit NF-κB translocation to the nucleus, but rather blocks NF-κB from binding to DNA and thereby its transcription activity (18, 38, 39). Previous studies suggested that the role of NFKBIZ varies in different cell types even under the same stimulation. For example, NFKBIZ promoted the release of inflammatory cytokines in macrophages but it inhibited the release of inflammatory cytokines in renal tubular cells upon LPS exposure (18, 39, 40). In our present study, knockdown of *Nfkbiz* increased the production of proinflammatory cytokines and tubular cell death during LPS treatment, suggesting a renoprotective role for NFKBIZ in septic AKI (Figure 8). These findings illustrate a negative feedback loop that limits NF-κB activation in renal tubular cells in septic AKI. Essentially, NF-κB is activated in sepsis to repress miR-376b, leading to the expression of NFKBIZ, which conversely inhibits NF-κB and associated inflammation and tubular injury, providing an intrinsic protective mechanism (Figure 10).

The mortality rate of patients with sepsis and AKI is markedly higher than that of patients with sepsis without AKI. As such, early, sensitive and specific diagnosis of AKI in these patients is critically important for decision-making and treatment in clinical settings. Obviously, this depends on the discovery of biomarkers with high sensitivity and specificity for early diagnosis of septic AKI (6). Serum creatinine and urine output are still the most widely used parameters for AKI diagnosis and staging in clinical practice (22). However, both parameters are affected by many factors besides kidney injury, and their changes often occur late after detectable kidney injury (22). Recently, protein biomarkers of AKI, such as kidney injury molecule 1 (KIM1) and neutrophil gelatinase–associated lipocalin (NGAL), have been discovered (22). These protein biomarkers are mainly based on renal tubular injury and are useful for specific subsets of patients with AKI. Compared with ischemic and nephrotoxic AKI, septic AKI is unique in that tubular cell injury and death is relatively mild, and renal tubule-derived protein biomarkers might not be detectable or sensitive enough (32). More recently, urinary TIMP2 and IGFBP7 were identified as biomarkers for AKI, and NephroCheck, based on the arithmetic product of the urinary concentrations of TIMP2 and IGFBP7, has been approved by the US FDA as a biomarker test for AKI diagnosis (1, 2, 32) The increase of urinary TIMP2 and IGFBP7 in AKI mainly results from increased glomerular filtration and decreased tubular reabsorption, but sepsis or endotoxemia does not increase glomerular filtration or induce overt tubular damage, making urinary TIMP2 and IGFBP7 less sensitive in detecting septic AKI (32). Recent studies suggested that serum and urinary miRNAs could serve as effective biomarkers for kidney diseases (25, 41). Compared with protein biomarkers, miRNAs are much more stable and PCR-based amplification ensures their high sensitivity and specificity (25). In our present study, patients with sepsis with or without AKI showed similar levels of miR-376b in serum. However, patients with sepsis and AKI had significantly lower miR-376b in urine than did patients with sepsis without AKI (Figure 9C). In addition, there was a significant negative correlation of urinary miR-376b with the levels of serum creatinine and BUN in patients with sepsis (Figure 9, D and E). Moreover, compared with urinary [TIMP2]*[IGFBP7], urinary miR-376b even showed a moderately higher sensitivity (65% vs. 60%) for detecting AKI in patients with sepsis (Table 2). Collectively, these data indicate that urinary miR-376b may be useful for AKI diagnosis in patients with sepsis.

However, there are several defects for urinary miR-376b as a biomarker for septic AKI. First, this is a single-center study with a relatively small sample size. It is important to further determine the diagnostic efficiency of miR-376b for septic AKI in a large cohort, multiple-center study. Second, the decrease of urinary miR-376b was not detected at the earliest time point of 4 hours after LPS treatment (Figure 9B), indicating that it may not serve as a predictive biomarker for septic AKI. In this aspect, urinary [TIMP2]*[IGFBP7] that was approved by the FDA to predict stage 2–3 AKI has an obvious advantage. Finally, we did not examine urinary miR-376b during the treatment and recovery in septic patients with AKI so it remains unclear whether miR-376b is an effective biomarker for prognosis and therapeutic efficacy. Therefore, our test of miR-376b is still at the discovery phase, and further research in animal models and patients with sepsis is required to validate the initial observation and examine its biomarker potential.

In conclusion, this study demonstrates NF-κB–mediated downregulation of miR-376b in renal tubular cells in septic AKI. Notably, miR-376b downregulation leads to the induction of NFKBIZ, forming a negative feedback loop to limit NF-κB activation, inflammation, and tubular cell death. This mechanism presents therapeutic targets for septic AKI. Moreover, the decrease in urinary miR-376b may be a useful biomarker for the diagnosis of AKI in sepsis.

## Methods

### Antibodies and special reagents.

Antibodies in the present study were from the following sources. Anti-p65 (8242), anti-p-p65 (3033), anti-cleaved caspase-3 (9664), and anti-GAPDH (5174) were from Cell Signaling Technology. Anti-NFKBIZ (14014) was from Proteintech. All secondary antibodies for immunoblot analysis were from Thermo Fisher Scientific. FITC- or rhodamine-conjugated goat anti-rabbit IgG were from Abcam. Special reagents were from the following sources: LPS was from MilliporeSigma; TPCA-1 (A4602) was from APExBIO; digoxigenin-labeled mmu–miR-376b LNA probes and the Fluorescence In Situ Hybridization Kit were from Servicebio; anti-DIG-HRP was from Jackson ImmunoResearch Inc., Human/mouse Tissue inhibitor of TIMP2 and human/mouse IGFBP7 ELISA Kits were from CUSABIO; miR-376b mimic was from Life Technologies; anti–miR-376b LNA, NC oligonucleotide LNA, and *Nfkbiz* siRNAs were from Ribo Biotechnology; Lipofectamine 2000 was from Thermo Fisher Scientific; and the Chromatin immunoprecipitation assay kit (ab500) was from Abcam.

### Animals and septic AKI induction.

Male C57BL/6 mice (8~10 weeks) were purchased from Hunan Slack King Experimental Animal Company. Septic AKI was induced by LPS injection or by CLP. For LPS induction, 1 dose of LPS (10 mg/kg body weight) was intraperitoneally injected into mice, while control mice were injected with normal saline. CLP surgery was performed as previously described (42). Briefly, mice were anesthetized with 60 mg/kg pentobarbital and a small longitudinal midline incision was made to expose the cecum to be ligated at 1 cm away from the blind-ending of caecum. A single through-and-through puncture midway between the ligation and the tip of the cecum in a mesenteric-to-antimesenteric direction was performed to perforate the cecum. After removing the needle, a small amount (droplet) of feces from both the mesenteric and antimesenteric penetration holes were extruded to ensure patency. The sham operation had the same procedure but without ligation and perforation (42). In some experiments, miR-376b mimic (7mg/kg), anti–miR-376b LNA (20 mg/kg), or NC oligonucleotide LNA were delivered to mice with Invivofectamine 2.0 (Life Technologies) through tail vein injection as described before (43–45).

### Cell cultures and chemical treatment.

The BUMPT was originally provided by Wilfred Lieberthal (Boston University School of Medicine, Boston, Massachusetts, USA). The cells were cultured in DMEM medium containing 10% FBS. For LPS treatment, BUMPT cells at a 50%–60% confluence were maintained in DMEM medium containing 0.2% FBS for 24 hours. The cells were then changed to DMEM medium containing 0.2% FBS plus 10 μg/mL LPS and cultured for another 8 hours. For TPCA-1 treatment, 100 μM TPCA-1 was added concurrently with LPS. In some experiments, 200 nM microRNA mimic, NFKBIZ siRNA, or NC oligonucleotides were transfected into BUMPT cells with Lipofectamine 2000 following the manufacturer’s instructions (43–45).

### Renal histology and TUNEL assay.

Kidney tissues were fixed with 4% paraformaldehyde and embedded in paraffin. Kidney tissue sections of 4 μm were then prepared. H&E staining was conducted to analyze renal histology. TUNEL assay in kidney tissue sections or cultured cells was performed as previously described (44). The slides were imaged with fluorescent microscopy and the TUNEL-positive cells were counted from 10 randomly picked images.

### Analysis of serum and urinary samples.

Serum creatinine and BUN were measured to evaluate renal function decline with reagents from BioAssay Systems. Urinary TIMP2 and IGFBP7 were measured by using ELISA Kits (CUSABIO) according to the manufacturer’s instructions.

### MicroRNA microarray analysis.

MicroRNA microarray was conducted as previously described (43–45). Briefly, total RNAs were extracted from the kidney cortexes of mice with or without LPS treatment. The RNA samples were reversely transcribed using the TaqMan MicroRNA Reverse Transcription Kit (Applied Biosystems). The product of reverse transcription was preamplified and then the microRNA expression was profiled with TaqMan Rodent MicroRNA array card A v2.0 following the manufacturer’s instructions. The global normalization process included the subtraction of the mean CT value of the reference set from the CT value of each microRNA of the same sample. Quantification of each sample was shown as 2-ΔΔCt values.

### qPCR.

For qPCR analysis of miRNAs, total RNAs were isolated from cultured cells and kidney tissues with the mirVana kit (Ambion), while total RNAs from urine and serum were prepared with the miRNeasy Serum/Plasma Kit (QIAGEN). 50 ng of total RNAs from each sample were reversely transcribed into cDNA by using the microRNA Reverse Transcription kit (Applied Biosystems), and qPCR analysis of target miRNA was performed by using the TaqMan microRNA assay kit (Applied Biosystems). For qPCR analysis of mRNAs, total RNAs were isolated from the kidney tissues or cultured cells using TRIzol (Thermo Fisher Scientific). 1 μg of RNAs from each sample were reversely transcribed into cDNA by using the M-MLV Reverse Transcriptase cDNA Synthesis Kit (TaKaRa Bio USA). qPCR was performed by using the SYBR Premix Ex Taq TM II (TaKaRa Bio USA). All PCR data were analyzed by the LightCycler 96 SW 1.1 software, and each sample was shown as 2-ΔΔCt values.

### Immunoblot analysis.

Immunoblot analysis was performed as previously described (46). Briefly, cells or kidney tissues were lysed with 2% SDS buffer containing protease inhibitor cocktail (MilliporeSigma, P8340). Equal amount of total proteins from different samples were separated on SDS-polyacrylamide gels and then transferred onto polyvinylidene difluoride membrane. The membrane was probed subsequently with primary antibodies and corresponding horseradish peroxidase–conjugated secondary antibodies. Antigen-antibody complex was detected with an enhanced chemiluminescence kit (Thermo Fisher Scientific, 32106).

### Immunofluorescence.

Paraffin-embedded kidney sections were sequentially subjected to deparaffinization, hydration, and antigen retrieval by incubation with 0.1 M sodium citrate, pH 6.0, at 100°C. Kidney sections or fixed cells were then permeabilized with 0.1% Triton X-100 and incubated in blocking buffer. The specimens were sequentially incubated with NFKBIZ (Proteintech, 14014, 1:200) at 4°C overnight, FITC- or rhodamine-conjugated secondary antibodies for 1 hour at room temperature, and DAPI (MilliporeSigma, D9542). All samples were examined by laser scanning confocal microscopy.

### Apoptosis analysis following Annexin V-FITC/PI staining.

Apoptosis in cultured cells was measured by flow cytometry after Annexin V/PI staining using the eBioscience Annexin V-FITC Apoptosis Detection Kit (Invitrogen, BMS500FI-100). Briefly, cells were harvested at indicated time points and washed in PBS by gentle shaking followed by incubation in 200 μL Binding Buffer that contained 5 μL Annexin V-FITC in dark for 10 minutes at room temperature. Then, the cells were washed in 200 μL Binding Buffer and further suspended in 190 μL Binding Buffer. 10 μL PI (20 μg/mL) was then added to the samples before analysis on a FACSCalibur flow cytometer within 1 hour.

### ChIP assay.

ChIP assay was performed as described in our previous work with minor modifications (43–45). The cells were fixed with 1% formaldehyde and then neutralized with glycine. The cell samples were then sonicated to shear the DNA. After that, the supernatants containing DNA were collected. Equal amounts of DNA from different samples were incubated with equal amount of anti-p65 antibody. The resultant immunoprecipitate was subjected to qPCR amplification of putative NF-κB binding sequences using specifically designed primers. The value of qPCR was normalized with input DNA for comparison. The following primers were used for NF-κB binding site detection: binding site 1, forward, GGCTGTGTGACAACCAGGG, and reverse, CCAGAGGCATAGAGGAGCAC; binding site 2, forward, GCCTGTCTGCTGCCTTCTTC, and reverse, AGCTCAGGCCCATTACCCAG; and binding site 3, forward, AGTGTCGGTACTGGTATGCCT, and reverse, TCACCACACTACCAGGTCCTT.

### Luciferase microRNA target reporter assay.

The target reporter assay was conducted as before (43–45). The 3′-UTR of the mouse *Nfkbiz* gene was synthesized and inserted at the 3′-UTR of the luciferase gene in the pMIR-REPORT Luciferase plasmid (Life Technologies). The plasmids were cotransfected with pMIR-REPORT β-gal control plasmid (Life Technologies) and 200 nM miR-376b mimics (Life Technologies) into HEK293 cells. One day after the transfection, cell lysate was collected in reporter lysis buffer from the Luciferase Assay System (Promega). The luciferase activity was detected by microplate reader and then normalized with β-galactosidase activity.

### In situ hybridization analysis of microRNA.

In situ hybridization was performed to localize microRNA expression in kidney tissues as described in our previous work (43–45). Briefly, after deparaffinization and hydration, paraffin-embedded kidney tissue sections were sequentially incubated with 20 μg/mL proteinase K for permeabilization, and prehybridization solution at 78°C for 1 hour. The tissues sections were then exposed to digoxigenin-labeled mmu–miR-376b LNA probe (Servicebio) at 37°C overnight. After incubation with 5% BSA to remove nonspecific staining, the tissue sections were probed with anti-digoxigeni-HRP at 37°C for 1 hour. The signal was revealed by adding DAB solution and recorded by microscopy.

### Patients and sample collection.

Serum and urine samples were collected from 10 healthy subjects, 20 patients with sepsis without AKI, and 20 patients with sepsis and AKI. Sepsis was diagnosed according to the International Sepsis Definition Conference criteria (47), and AKI was diagnosed according to the KDIGO criteria (48). Patients with CKD or neoplastic diseases were excluded from the study. Basic information of the participants is listed in Supplemental Table 1. Urine and blood samples were collected and centrifuged at 850*g* for 10 minutes to collect the supernatants for analysis.

### Statistics.

Statistical differences between the 2 groups were analyzed by 2-tailed Student’s *t* test, and differences in multiple groups were determined by 1-way ANOVA analysis. ROC analysis and logistic regression were applied to evaluate the efficiency (sensitivity and specificity) of diagnostic indicators. Pearson correlation analysis was performed to evaluate the strength of the relationship between 2 quantitative variables. Data are expressed as the mean ± SD. *P* < 0.05 was considered significant. GraphPad Prism 7.0 and SPSS 22.0 were applied for statistical analysis.

### Study approval.

Animal experiments were conducted in accordance with a protocol approved by the Institutional Animal Care and Use Committee of the Second Xiangya Hospital of Central South University and the NIH *Guide for the Care and Use of Laboratory Animals* (National Academies Press, 2011). The human study was approved by the ethics committee of the Second Xiangya Hospital of Central South University. Before inclusion, all participants in the study were recruited with written informed consent.

## Author contributions

ZD, DZ, and ZL designed this study; ZL performed most of the experiments; DY, JZ,SS, YL, and LY contributed to the collection of human patient samples and clinical data; LH and JC contributed to data analysis; GC,YL, and CT provided suggestions for manuscript preparation; ZD and DZ contributed to data analysis and manuscript preparation.

## Supplementary Material

Supplemental data

## Figures and Tables

**Table 1:**
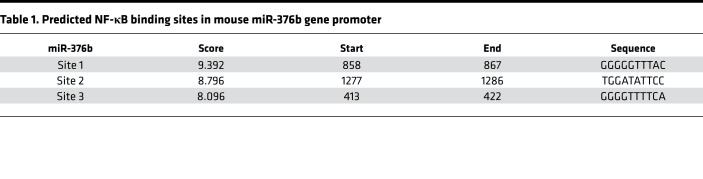
Predicted NF-κB binding sites in mouse miR-376b gene promoter

**Table 2:**
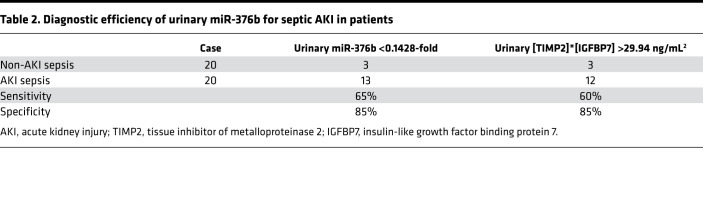
Diagnostic efficiency of urinary miR-376b for septic AKI in patients

**Table 3:**
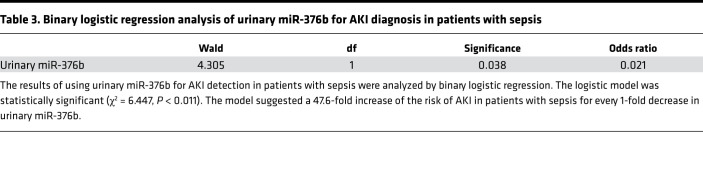
Binary logistic regression analysis of urinary miR-376b for AKI diagnosis in patients with sepsis

## Version 1 12/17/2020

Electronic publication
