# Supplementary material for: The negative feedback loop of NF-κB/miR-376b/NFKBIZ in septic acute kidney injury
Source: JCI Insight. 2020 Dec 17;5(24):e142272. doi: 10.1172/jci.insight.142272 (PMC7819752; doi:10.1172/jci.insight.142272)
Supplement: Supplemental data [file jciinsight-5-142272-s148.pdf]

Supplementary Data

5 Supplementary Figures

1 Supplementary Table

The Negative Feedback Loop of NFKB/miR-376b/NFKBIZ in Septic Acute Kidney Injury

Zhiwen Liu[1], Chengyuan Tang[1], Liyu He[1], Danyi Yang[1], Juan Cai[1], Jiefu Zhu[1], Shaoqun Shu[1], Yuxue Liu[1], Lijun Yin[1], Guochun Chen[1], Yu Liu[1], Dongshan Zhang[1,2]*, Zheng Dong[1,3]*

Table S1: Clinical test results of the participants.

	Control	No-AKI sepsis	AKI sepsis	P value
Gender(male/female)	4/6	13/7	8/12	0.12
Age (Years)	55.5±5.8	54.85 ± 4.11	55.20 ± 4.13	0.94
Creatinine, μmol/L	64.34±11.27	76.71 ± 7.25	345.20 ± 53.63	<0.01 *
Blood urea nitrogen, mmol/L	4.15±1.72	5.72 ± 0.67	26.24 ± 2.46	<0.01 *
Serum albumin, g/L	46.05 ± 2.4	25.72 ± 0.78	28.07 ± 0.96	0.07
Hemoglobin, g/L	122.1 ± 26.8	100.3 ± 4.81	100.2 ± 4.48	0.99
Neutrophils, $X10^9$	3.34 ± 1.46	13.48 ± 1.01	13.5 ± 1.07	0.98
Blood sedimentation		80.21 ± 6.81	58.71 ± 8.03	0.04 *
CRP, mg/L		187.2 ± 16.46	171.5 ± 34.72	0.68
Calcitonin original, ng/L		18.5 ± 6.75	23.09 ± 6.53	0.63

*P<0.05 versus Non-AKI sepsis. Values are Mean ± SEM.

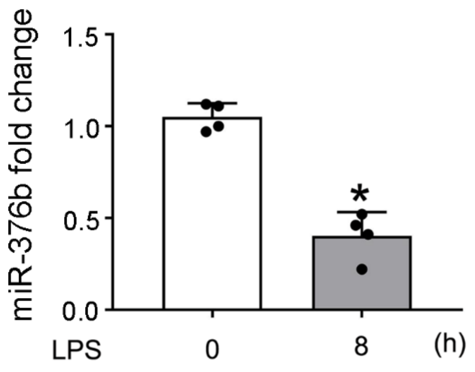

Figure S1: miR-376b is down-regulated by LPS in BUMPT cells. BUMPT cells were maintained in DMEM containing 0.2% FBS for 24 hours followed by treatment with 10μg/ml LPS in DMEM containing 0.2% FBS for 8 hours. miR-376b was quantified by Taqman assay-based qPCR and normalized to the level of sno202 (internal control) to determine the ratios with the ratios of control cells arbitrarily set as 1. Values are mean ± SD (n=4,2-tailed Student's t test),*p<0.05 vs control cells.

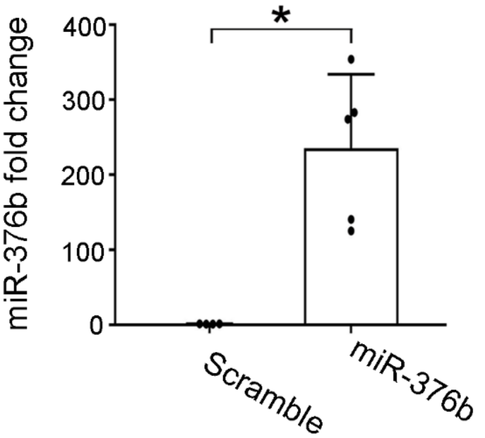

Figure S2: qPCR of miR-376b to verify successful RNA oligo delivery to mouse kidneys. miR-376b was quantified by Taqman assay-based qPCR and normalized to the level of sno202 (internal control) to determine the ratios with the ratios of negative control mice arbitrarily set as 1. Quantitative data are expressed as mean ± SD (n=4-5, 2-tailed Student's t test),*P<0.05.

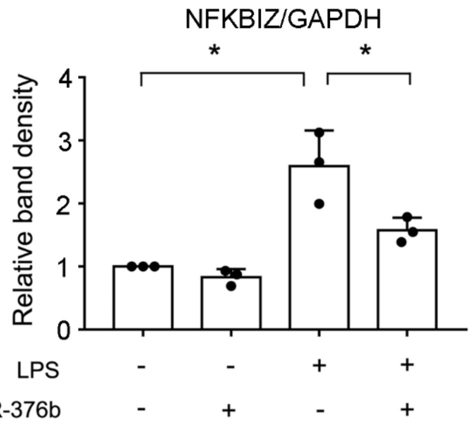

Figure S3: miR-376b targets NFKBIZ in LPS induced septic AKI. Semi-quantification of NFKBIZ. Protein bands in immunoblots were analyzed by densitometry to calculate the signal ratio of NFKBIZ/GAPDH with the ratio in control cells were arbitrarily set as 1. Data are expressed as mean ± SD (n=3, 1-way ANOVA with Tukey's multiple comparisons test), *P<0.05.

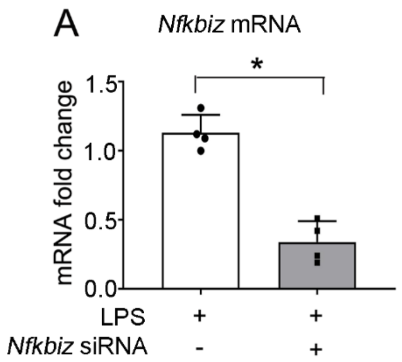

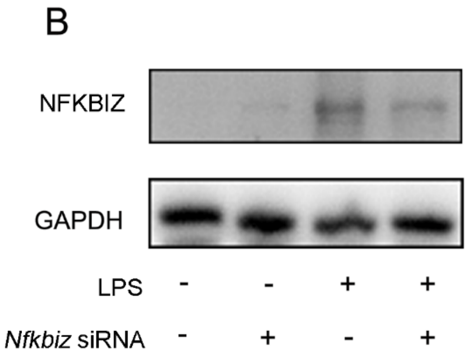

Figure S4: Verify the silencing effect of *Nfkbiz* siRNA after transfection. (A) qPCR to verify the silencing effect of *Nfkbiz* siRNA after transfection, Data are expressed as mean ± SD (n=4,2-tailed Student's t test,) *P<0.05. **(B)** Immunoblot to verify the silencing effect of *Nfkbiz* siRNA after transfection. GAPDH was used as a loading control.

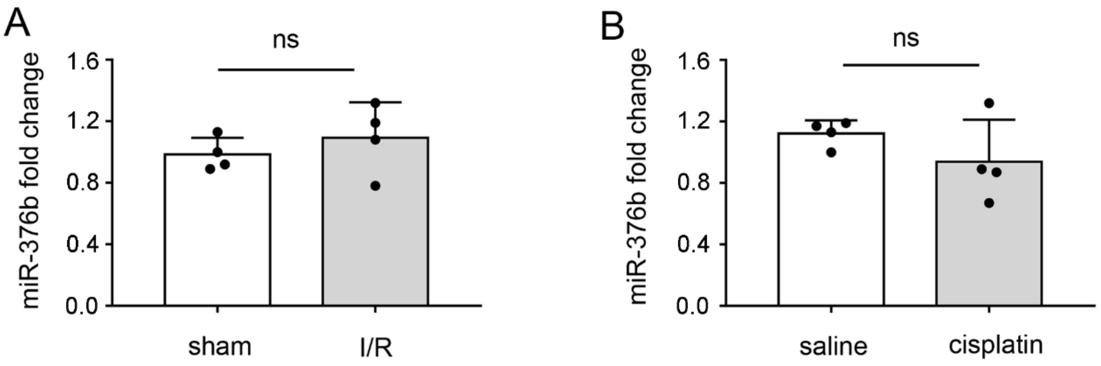

Figure S5: Changes in miR-376b expression in kidney tissues during ischemic AKI and cisplatin-induced nephrotoxic AKI in mice. (A) qPCR analysis showing miR-376b was not changed in ischemic AKI. The mice were subjected to 30 minutes of bilateral renal ischemia followed by 48 hours of reperfusion, or sham operation. (B) qPCR analysis showing miR-376b was not changed in cisplatin-induced nephrotoxic AKI. The mice were treated with 30mg/Kg of cisplatin or proper amount of saline (control) for 3 days. At indicated time point, the mice were sacrificed and kidney tissues were collected for qPCR analysis. The level of miR-376b was normalized to the level of sno202 of the same samples to determine the ratio with the ratio of control mice arbitrarily set as 1. Quantitative data are expressed as mean ± SD (n=4,2-tailed Student's t test). ns represents not statistically significant.
